# Antimicrobial activity and antibiotic susceptibility of *Lactobacillus* and *Bifidobacterium* spp. intended for use as starter and probiotic cultures

**DOI:** 10.1080/13102818.2014.987450

**Published:** 2014-12-11

**Authors:** Ralitsa Georgieva, Lyubomira Yocheva, Lilia Tserovska, Galina Zhelezova, Nina Stefanova, Akseniya Atanasova, Antonia Danguleva, Gergana Ivanova, Nikolay Karapetkov, Nevenka Rumyan, Elena Karaivanova

**Affiliations:** ^a^ Lactina Ltd., Bankya, Bulgaria; ^b^ Department of Biology, Medical Genetics and Microbiology, Faculty of Medicine, Sofia University “St. Kliment Ohridski”, Sofia, Bulgaria

**Keywords:** lactic acid bacteria, antagonistic effect, MIC

## Abstract

Antimicrobial activity and antibiotic susceptibility were tested for 23 *Lactobacillus* and three *Bifidobacterium* strains isolated from different ecological niches. Agar-well diffusion method was used to test the antagonistic effect (against *Staphylococcus aureus*, *Escherichia coli*, *Bacillus cereus* and *Candida albicans*) of acid and neutralized (pH 5.5) lyophilized concentrated supernatants (cell-free supernatant; CFS) and whey (cell-free whey fractions; CFW) from de Man–Rogosa–Sharpe/trypticase-phytone-yeast broth and skim milk. Acid CFS and CFW showed high acidification rate-dependent bacterial inhibition; five strains were active against *C. albicans.* Neutralized CFS/CFW assays showed six strains active against *S. aureus* (*L. acidophilus* L-1, *L. brevis* 1, *L. fermentum* 1, *B. animalis* subsp*. lactis* L-3), *E. coli* (*L. bulgaricus* 6) or *B. cereus* (*L. plantarum* 24-4В). Inhibition of two pathogens with neutralized CFS (*L. bulgaricus* 6, *L. helveticus* 3, *L. plantarum* 24-2L, *L. fermentum* 1)/CFW (*L. plantarum* 24-5D, *L. plantarum* 24-4В) was detected. Some strains maintained activity after pH neutralization, indicating presence of active substances. The antibiotics minimum inhibitory concentrations (MICs) were determined by the Epsilometer test method. All strains were susceptible to ampicillin, gentamicin, erythromycin and tetracycline. Four lactobacilli were resistant to one antibiotic (*L. rhamnosus* Lio 1 to streptomycin) or two antibiotics (*L. acidophilus* L-1 and *L. brevis* 1 to kanamycin and clindamycin; *L. casei* L-4 to clindamycin and chloramphenicol). Vancomycin MICs > 256 μg/mL indicated intrinsic resistance for all heterofermentative lactobacilli. The antimicrobially active strains do not cause concerns about antibiotic resistance transfer and could be used as natural biopreservatives in food and therapeutic formulations.

## Introduction

Foods are now considered not only in terms of taste and immediate nutritional needs, but also in terms of their ability to improve the health and well-being of consumers.[1] Hence, the increased interest in food ingredients with valuable bioactive properties and, consequently, in lactic acid bacteria (LAB) and bifidobacteria with antagonistic activity against pathogenic micro-organisms. There are different mechanisms for control and inhibition of other microbes, e.g. nutrient competition, production of inhibitory compounds, immunostimulation and competition for binding sites. Among these activities, the production of organic acids (such as lactic acid), which results in lowered pH, is the most important. Additionally, certain strains are also capable of producing bioactive molecules, such as ethanol, formic acid, fatty acids, hydrogen peroxide and bacteriocins, that have antimicrobial activity.[2] *Lactobacillus* and *Bifidobacterium* spp. and their by-products have been shown to be effective in several aspects. One of the most important advantages is the extended shelf life and safety of minimally processed foods, because these antimicrobial substances are safe and effective natural inhibitors of pathogenic and food spoilage bacteria in various foods. Additionally, the consumption of viable bacteria in the form of probiotics and functional foods is widely used for improvement of the balance and activity of the advantageous intestinal microflora, which has prophylactic benefit.[1]

The close contact with native microbiota in the human intestine is an excellent precondition for horizontal transfer of antimicrobial resistance genes with the aid of mobile genetic elements.[3] Therefore, the safety of cultures intended for use as food additives should be carefully re-assessed, even though most strains of the *Lactobacillus* and *Bifidobacterium* group are classified as ‘generally recognized as safe’ bacteria due to their long history of safe use and proven health benefits. Thus, antibiotic-resistance screening for starter and probiotic cultures now tends to become systematic. In order to eliminate the possibility of acquired resistance, the Panel on Additives and Products or Substances used in Animal Feed (FEEDAP) of the European Food Safety Authority (EFSA) requires the determination of the minimum inhibitory concentrations (MICs) of the most relevant antibiotics for each bacterial strain that is used as a feed additive.[4]

In this study, *Lactobacillus* and *Bifidobacterium* spp. were screened for their antagonistic activity against four food-borne and human pathogens and antibiotic susceptibility for development of probiotics and food biopreservatives.

## Materials and methods

### Bacteria and source of isolation

Twenty-three *Lactobacillus* strains (13 homofermentative and 10 heterofermentative) and three *Bifidobacterium* strains, part of the laboratory collection of Lactina Ltd. (Bankya, Bulgaria), were selected for this study. In a preliminary (unpublished) study, the strains were identified using biochemical (API 50 CHL) and molecular tests (species-specific polymerase chain reaction or sequence analysis). The source of isolation for each strain is presented in Table 1. All cultures were stored at −65 °C in appropriate broth media supplemented with glycerol (20% v/v). Before the assay, the strains were pre-cultivated twice in MRS (de Man–Rogosa–Sharpe) broth (Hi-Media Pvt. Ltd., India) for lactobacilli or TPY broth (trypticase-phytone-yeast) for bifidobacteria at 37 °C for 24 h.

**Table 1.** *Lactobacillus* and *Bifidobacterium* strains included in this study and source of isolation.

Strain	Source of isolation
*L. bulgaricus*: *L. b* 1; *L. b* 2; *L. b* 5; *L. b* 6; *L. b* 7E *L. plantarum*: *L. pl* 24-4B *L. brevis*: *L. br* 1	Home-made yoghurt
*L. casei*: *L. c* L-4 *L. rhamnosus*: *L. rh* Lio2 *L. paracasei*: *L. parac* 4k	Home-made cheese
*L. helveticus*: *L. h* N11; *L. h* N12; *L. h* 3 *L. lactis*: *L. lc* L-14	Yellow cheese whey
*L. acidophilus*: *L. a* 10	Plant origin-melon
*L. helveticus: L. h* AFA	Blue-green algae
*L. plantarum*: *L. pl* 24-5D	Pickle
*L. plantarum*: *L. pl* 24-2L; *L. pl* 24-3L	Raw-fermented sausages
*L. acidophilus: L. a* L-1 *L. helveticus*: *L. h* 108 *L. rhamnosus*: *L. rh* Lio1 *B. longum*: *B. lg* L-1 *B. bifidum*: *B. bf* L-2 *B. animalis* subsp. *lactis*: *B. lc* L-3	Baby faeces
*L. fermentum*: *L. f* 1	Saliva

### Test micro-organisms

Three bacterial food-borne pathogens and one yeast culture were selected as test micro-organisms and were obtained from the National Bank for Industrial Micro-organisms and Cell Cultures (Bulgaria): *Staphylococcus aureus* NBIMCC 3703, *Escherichia coli* NBIMCC 3702, *Bacillus cereus* NBIMCC 1085 and *Candida albicans* NBIMCC 74. The cultures of *S. aureus* and *E. сoli* were propagated in nutrient broth (NB, HiMedia), *B. cereus* in tryptic soy broth (TSB, Merck, Germany) and *C. аlbicans* in Sabouraud dextrose broth (HiMedia).

### Antimicrobial activity assay

Two model systems for antimicrobial production were applied: cultivation in MRS or TPY broth (for *Lactobacillus* and *Bifidobacterium* spp., respectively) and cultivation in 10% (w/v) skim milk (Fude + Serrahn Milchprodukte GmbH & Co, Germany). The media were inoculated with 10% (v/v) previously activated *Lactobacillus* or *Bifidobacterium* culture. After incubation at 37 °C for 28 h, the cultures were centrifuged (5000 *g* for 20 min at 5 °C) for removal of bacterial cells. Part of the cell-free supernatants (CFS) and the cell-free whey fractions (CFW) were left with their initial acid pH. The rest of the samples were buffered with 5 mol/L NaOH at рН 5.5 ± 0.1 in order to eliminate the putative effect of produced organic acids. The pH values of the neutralized samples were consistent with the pH of LAB cultures before freeze drying in the real technological process. After filtration (0.22 μm pore size; Millipore), the acid and neutralized CFS (aCFS and nCFS) and CFW (aCFW and nCFW) were lyophilized (Martin Christ GmbH, Germany) in Petri dishes (10 mL) at the following conditions: freezing at −45 °С for 2 h, heating at 32 °C, vacuum 0.370 mbar, duration 40 h. The obtained dry samples were dissolved in 2 mL of sterile distilled water (resulting in 5× concentration increase as compared to the initial culture prior to lyophilization) and stored at −65 °C until later use in the screening procedures.

Аgar-well diffusion method was used to determine the inhibitory effect.[5] Exponential cultures of the test micro-organisms were diluted to a suitable turbidity and used to inoculate a melted and cooled Mueller–Hinton Agar (MHA, HiMedia) to a final concentration of ∼10^6^–10^7^ CFU/mL. Only *C. albicans* was plated on Sabouraud dextrose agar (HiMedia) by spreading the cell suspension with a sterile cotton swab. Wells, 8 mm in diameter, were punched in the agar plates and 100 μL of CFS and CFW were added to the wells. After incubation overnight at 37 °C, the antimicrobial activity was expressed as the diameter of the inhibition zones (mm) around the wells. Zones of inhibition ≥10 mm were regarded as positive.

### Antibiotic susceptibility

For selected *Lactobacillus* and *Bifidobacterium* strains, the MICs (μg/mL) of nine antibiotics were determined using commercial E-test® (Epsilometer test, bioMerieux, France): ampicillin, vancomycin, gentamicin, kanamycin, streptomycin, erythromycin, clindamycin, tetracycline and chloramphenicol. The concentration on the strips was from 0.016 to 256 μg/mL with the exception of streptomycin (0.064–1024 μg/mL). Bacterial cultures in the exponential growth phase were diluted to a suitable turbidity and used to inoculate a melted and cooled iso-sensitest agar (90% w/v, Oxoid, UK) supplemented with MRS or TPY agar (10% w/v) [6] to a final concentration of ∼10^6^–10^7^ CFU/mL. E-test strips were placed on the surface of the inoculated agar and incubated at 37 °C for 24 h. The MIC was interpreted as the point at which the ellipse intersected the E-test strip as described in the E-test technical guide.

## Results and discussion

Antimicrobial activity is a very important criterion for selection of starter and probiotic culture as natural antagonists of potentially harmful bacteria. Therefore, 23 *Lactobacillus* and three *Bifidobacterium* strains from the Lactina Ltd. collection were screened for their activity against four food-borne and human pathogens: *Staphylococcus aureus*, *Escherichia coli*, *Bacillus cereus* and *Candida albicans.* Lyophilized and concentrated, acid and neutralized cell-free filtrates obtained after cultivation of the selected lactobacilli and bifidobacteria in MRS or TPY broth (CFS) and skim milk (CFW) were tested for activity. Skim milk was chosen as a second model system because it is a natural medium for the growth of most LAB and bifidobacteria and is commonly used for production of freeze-dried cultures. At the same time, it is an excellent medium for development of many pathogens.

**Figure 1.**
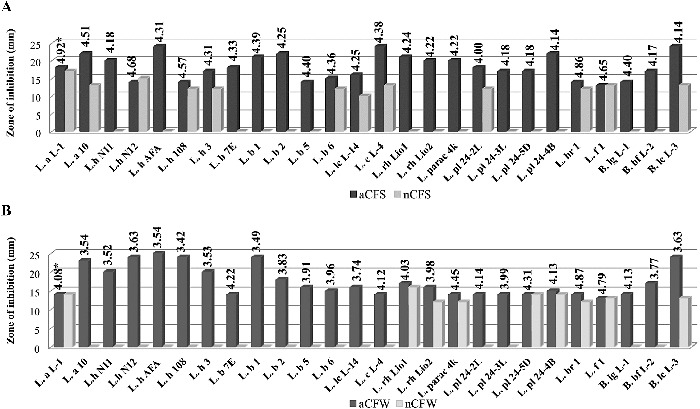
Antimicrobial activity of *Lactobacillus* and *Bifidobacterium* strains against *Staphylococcus aureus* NBIMCC 3703: (A) aCFS and nCFS; (B) aCFW and nCFW. *pH values of acid CFSs and acid CFWs.

**Figure 2.**
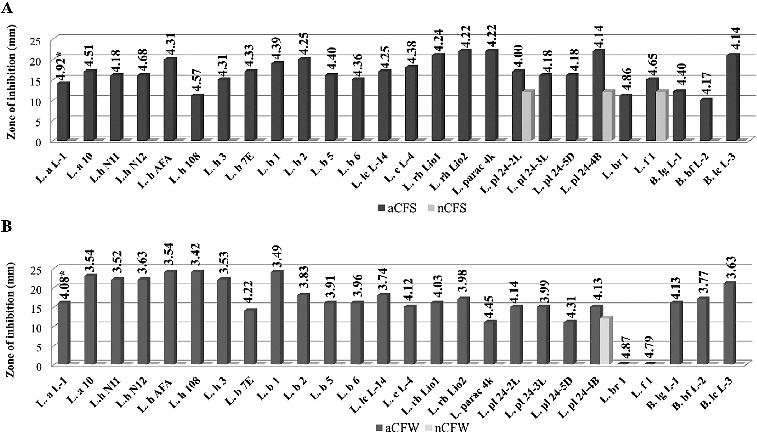
Antimicrobial activity of *Lactobacillus* and *Bifidobacterium* strains against *Bacillus cereus* NBIMCC 1085: (A) aCFS and nCFS; (B) aCFW and nCFW. *pH values of acid CFSs and acid CFWs.

**Figure 3.**
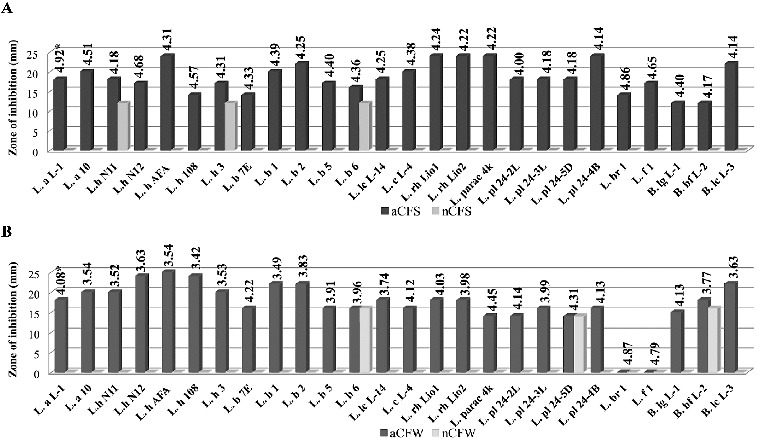
Antimicrobial activity of *Lactobacillus* and *Bifidobacterium* strains against *Escherichia coli* NBIMCC 3702: (A) aCFS and nCFS; (B) aCFW and nCFW. *pH values of acid CFSs and acid CFWs.

Acid CFSs and CFWs of all tested cultures showed activity against *S. aureus*, *B. cereus* and *E. coli* (Figures 1–3). Correlation between the rate of acidification (pH) and the diameter of inhibitory zone was observed for most strains. The aCFWs of two strains, *L. brevis* 1 (рН 4.87) and *L. fermentum* 1 (рН 4.79), were inhibitory only for *S. aureus*. *C. albicans* was less affected. Acid CFSs of only five strains (*L. bulgaricus* 1, *L. bulgaricus* 2, *L. rhamnosus* Lio1, *L. paracasei* 4K, *L. plantarum* 24-4В) were active against the yeast. None of the acid CFWs inhibited *C. albicans* (data not shown).

After pH neutralization, 18 strains were determined as active due to the observed ability to inhibit the growth of at least one target strain. In most cases, a bacteriostatic zone of inhibition was observed. The highest activity with nCFSs and nCFWs was registered against *S. Aureus*: 46.2% and 35.6% of the strains, respectively. Higher activity with nCFSs was observed among the strains of *L. acidophilus*, *L. bulgaricus*, *L. helveticus* and *L. lactis* (Figure 1(A)). Conversely, nCFWs of *L. plantarum*, *L. paracasei*, *L. rhamnosus* and *L. fermentum* strains with antistaphylococcal activity were predominant (Figure 1(B)). Three strains with nCFS and one strain with nCFW were active against *B. cereus* (Figure 2). Аlso three strains with nCFS and nCFW inhibited *E. coli* (Figure 3). Six strains were active against *S. aureus* (*L. acidophilus* L-1, *L. brevis* 1, *L. fermentum* 1 and *B. animalis* subsp*. lactis* L-3), *E. coli* (*L. bulgaricus* 6) or *B. cereus* (*L. plantarum* 24-4В) in both model systems (broth and milk). Inhibition of two pathogens was also observed. Activity against both Gram-positive micro-organisms *S. aureus* and *B. cereus* showed nCFS of *L. plantarum* 24-2L and *L. fermentum* 1, and nCFW of *L. plantarum* 24-4В. Activity against *S. aureus* and *E. coli* showed nCFS of *L. bulgaricus* 6 and *L. helveticus* 3, and nCFW of *L. plantarum* 24-5D. Activity of nCFSs and nCFWs against the three bacterial test micro-organisms was not registered. None of the tested nCFSs and nCFWs was active against *C. аlbicans* (data not shown).

The obtained results clearly show the role of acidity and pH for the antagonistic activity of *Lactobacillus* and *Bifidobacterium* spp. *in vitro*. The increased production of lactic acid through fermentation reduces pH of the media, which is known to inhibit the growth of most food-borne pathogens. The antimicrobial effect is also due to the undissociated form of the acid and its capacity to reduce the intracellular pH, leading to inhibition of vital cell functions.[7] Different sensitivity of the test micro-organisms determines different zone of inhibition at the same pH. The lack of activity against *E. coli* for two strains with aCFW could be explained with the results obtained by Goel et al.[8] for increased survival of *E. coli* in a fermented milk product with pH over 4.6.

The observed inhibition for some strains after elimination of the putative effects of lactic acid raised the question for possible production of other inhibitor substances, such as hydrogen peroxide, bacteriocin and bacteriocin-like substances. The greater activity against Gram-positive micro-organisms than against Gram-negative ones that was observed in our work is in accordance with the previous studies.[7] The activity against Gram-positive pathogens is mostly due to the bactericidal effect of protease sensitive bacteriocins,[2,9] while the antagonistic effects towards Gram-negative pathogens could be related to the production of organic acids and hydrogen peroxide.[10,11] However, a few bacteriocins of LAB active against *E. coli* and *Salmonella typhimurium* have also been reported.[12,13] On the other hand, the antibacterial activity of six strains (*L. acidophilus* L-1, *L. bulgaricus* 6, *L. plantarum* 24-4В, *L. fermentum* 1, *L. brevis* 1 and *B. animalis* subsp. *lactis* L-3) in both system (broth and milk) suggests a mechanism of action different from that mentioned above. The application of such strains gives a potential advantage in the food preservation strategy.

Regardless of the nature of the antibacterial substances produced by the neutralized variants, the ability to retain this activity after lyophilization would allow production of active dry starter and probiotic cultures. Strains *L. plantarum* 24-2L and 24-4B and *L. fermentum* 1 could be used as starter organisms in the production of bread and bakery products due to their activity against *B. cereus*. The inhibitory effect of *Lactobacillus* strains used as starters against rope-forming *Bacillus* has been previously reported.[14,15] Lactobacilli have also been shown to be effective in preventing the recurrence of urinary tract infection in women,[16] and traveler's diarrhea.[17] *E. coli* is the most common cause of these diseases. In this aspect, *L. bulgaricus* 6 and *L. helveticus* N11 exhibiting activity against this pathogen are good candidates for alleviating the symptoms and prophylaxis of such conditions. In our previous study,[18] *L. helveticus* N11 was proved to be active against the uropathogenic *E. coli* strain 536 and enteropathogenic *E. coli* strain E2348. Use of strains inhibiting *S. aureus* and *E. coli* as antimicrobial agents may provide a safe alternative in food preservation. A few studies reported *Lactobacillus* spp. with strong anti-Candida activity.[9,19] Although there are some clinical trials that support the effectiveness of lactobacilli for prevention or treatment of vaginal yeast infections (*C. albicans*), evidence regarding potential benefit still remains inconclusive.[20] The presence of active strains with potential application as natural biopreservatives or as probiotic cultures in specific therapeutic formulas determined our next steps towards more profound examination of the nature of the antimicrobial substances produced by selected *Lactobacillus* and *Bifidobacterium* strains.

In addition to antimicrobial activity, the MICs of nine antimicrobials of human and veterinary importance were determined for all strains. Lack of transferable resistance against therapeutic antibiotics is an important criterion for selection of an appropriate functional strain.[4] Two groups of antibiotics are generally recommended: inhibitors of cell-wall synthesis (ampicillin and vancomycin) and inhibitors of protein synthesis (chloramphenicol, gentamicin, streptomycin, kanamycin, tetracycline, erythromycin and clindamycin). The obtained results and reference microbiological breakpoints are presented in Table 2. A micro-organism inhibited at breakpoint level to a specific antimicrobial is defined as susceptible. When the MIC is higher than the breakpoint, the micro-organism is considered resistant.[4] For the analysis, E-test was chosen in our study, as it is a simple quantitative method that is commonly used for antimicrobial susceptibility testing of different micro-organisms.[21–23]

**Table 2.** MIC (μg/mL) of antimicrobials for *Lactobacillus* and *Bifidobacterium* strains determined by E-test®.

	Susceptibility to the following antibiotic MIC (μg/mL)								
Tested strain	AM	VM	GM	KM	SM	EM	CM	TC	CL
EFSA L. acidophilus group*	**1**	**2**	**16**	**64**	**16**	**1**	**1**	**4**	**4**
*L. a* L-1	0.125	1	12	>256^R^	6	0.75	4^R^	0.75	1.5
*L. a* 10	0.19	0.19	0.25	8	0.50	0.032	0.19	0.75	1.5
EFSA obligate homofermentative*	**1**	**2**	**16**	**16**	**16**	**1**	**1**	**4**	**4**
*L. h* N11	1	0.50	2	4	6	0.25	0.50	1	3
*L. h* N12	0.094	0.094	1.5	12	0.50	0.064	0.125	1	2
*L. h* AFA	0.047	0.094	0.094	12	0.50	0.064	0.19	0.19	2
*L. h* 108	0.032	0.38	0.75	12	0.75	0.023	0.50	0.75	0.032
*L. h* 3	0.023	1	2	16	4	0.50	0.75	1	2
*L. b* 7E	0.19	0.25	0.75	6	12	<0.016	0.032	0.125	4
*L. b* 1	0.023	0.38	1	8	4	<0.016	0.023	0.50	4
*L. b* 2	0.047	0.38	0.75	6	4	< 0.016	0.023	0.50	3
*L. b* 5	0.064	0.75	1.5	6	4	<0.016	0.016	0.50	3
*L. b* 6	0.125	0.38	1	8	6	< 0.016	0.023	0.25	4
*L. lc* L-14	0.094	0.25	2	12	12	<0.016	0.047	0.75	0.094
EFSA L. casei/paracasei*	**4**	n.r.	**32**	**64**	**64**	**1**	**1**	**4**	**4**
*L. c* L-4	0.19	>256	<0.016	12	4	0.19	2^R^	1	8^R^
*L. parac* 4k	0.50	>256	0.19	32	32	0.125	<0.016	<0.016	3
EFSA L. rhamnosus*	**4**	n.r.	**16**	**64**	**32**	**1**	**1**	**8**	**4**
*L. rh* Lio1	0.75	>256	0.75	12	>1024^R^	1	0.75	0.50	4
*L. rh* Lio2	0.19	>256	<0.016	4	4	0.023	0.19	0.19	3
EFSA L. plantarum/pentosus*	**2**	n.r.	**16**	**64**	n.r.	**1**	**2**	**32**	**8**
*L. pl* 24-2L	0.38	>256	3	32	24	0.75	2	16	4
*L. pl* 24-3L	0.75	>256	4	48	32	1	2	24	4
*L. pl* 24-5D	1	>256	4	48	64	0.75	0.75	24	6
*L. pl* 24-4B	0.75	>256	3	48	48	0.75	1	16	4
EFSA obligate heterofermentative*	**2**	n.r.	**16**	**32**	**64**	**1**	**1**	**8**	**4**
*L. br* 1	0.38	>256	2	64^R^	16	0.19	12^R^	6	4
*L. f* 1	0.023	>256	0.38	12	4	0.38	<0.016	0.38	1.5
EFSA Bifidobacterium*	**2**	**2**	**64**	n.r.	**128**	**1**	**1**	**8**	**4**
*B. lg* L-1	<0.016	0.50	2	12	2	0.023	0.023	1	3
*B. bf* L-2	<0.016	0.38	2	8	2	0.023	0.023	0.75	2
*B. lc* L-3	0.047	0.75	3	12	4	0.064	0.50	1	3

Note: AM – ampicillin, VM – vancomycin, GM – gentamicin, KM – kanamycin, SM – streptomycin, EM – erythromycin, CM – clindamycin, TC – tetracycline, CL – chloramphenicol.

*Strains with MIC higher than the breakpoints are considered as resistant (R) according to EFSA.[4].

n.r. – not required.

The bold values are reference values given by EFSA. [4] and that is why they are visually emphasized. This would allow easier comparison with the values obtained for the tested strains.

In this study, all tested *Lactobacillus* and *Bifidobacterium* strains were susceptible toward ampicillin, gentamicin, erythromycin and tetracycline (Table 2). For most of the strains kanamycin, clindamycin, streptomycin and chloramphenicol were effective inhibitors. Only four lactobacilli could be considered resistant to one antibiotic (*L. rhamnosus* Lio 1 to streptomycin) or two antibiotics (*L. acidophilus* L-1 and *L. brevis* 1 to kanamycin and clindamycin, *L. casei* L-4 to clindamycin and chloramphenicol) with MICs higher than the breakpoints recently proposed by the FEEDAP Panel.[4]

The obtained results are in accordance with previously reported data for lactobacilli and bifidobacteria. Generally, they are sensitive to the Gram-positive spectrum antibiotic erythromycin, the broad-spectrum antibiotics tetracycline and chloramphenicol and the beta-lactam antibiotic ampicillin.[21,23,24] Nevertheless, acquired genes which are potentially transferable have been detected in lactobacilli.[25] Among the most commonly observed resistance genes, there are two genes coding for tetracycline and erythromycin resistance, followed by genes for chloramphenicol resistance.[26,27] Thus, the chloramphenicol resistance of one of the *Lactobacillus* strains tested in our study deserves special attention in order to avoid potential risk. By contrast, the resistance against Gram-negative spectrum antibiotics kanamycin and streptomycin is frequently observed in lactobacilli and bifidobacteria.[6,21–23] It may be explained by the high rate of spontaneous chromosomal mutations conveying resistance to these antibiotics.[23,28] Strains with this type of acquired resistance have a low potential for horizontal spread and may be used as feed additives.[4] Among the aminoglycosides, lower MIC for gentamicin compared to kanamycin and streptomycin was observed as previously reported by Danielsen and Wind.[23] Although clindamycin is one of the most effective antibiotics against Gram-positive micro-organisms, three of the tested lactobacilli (*L. casei* L-4, *L. acidophilus* L-1 and *L. brevis* 1) were shown to be resistant according to the microbiological breakpoint of this drug. Clindamycin is used for treatment of bacterial vaginosis and resistant strains could be used to restore the normal vaginal microflora together with antimicrobial bacterial vaginosis treatment.[29]

*L. acidophilus*, *L. helveticus*, *L. bulgaricus*, *L. lactis* and *Bifidobacterium* proved to be very susceptible to vancomycin, as reported by other authors.[6,21] However, the highest concentration of this antibiotic was not inhibiting for all heterofermentative lactobacilli (Table 2). This resistance was previously documented as intrinsic or ‘natural’.[6] According to EFSA,[4] bacterial strains carrying intrinsic resistance present a minimal risk for horizontal spread and thus, may be used as a feed additive.

## Conclusions

This study tested the antimicrobial activity and antibiotic susceptibility of 26 *Lactobacillus* and *Bifidobacterium* strains. The results obtained at a laboratory scale allowed selection of active strains. Ten strains with antimicrobial activity against two pathogens or in both model systems (broth and milk) appeared to be most promising: *L. acidophilus* L-1; *L. bulgaricus* 6; *L. helveticus* N11; *L. helveticus* 3; *L. plantarum* 24-2L; *L. plantarum* 24-4В; *L. plantarum* 24-5D; *L. fermentum* 1; *L. brevis* 1 and *B. animalis* subsp*. lactis* L-3. They may play an important role in the food industry as starter cultures, co-cultures or bioprotective cultures, to improve food quality and safety or as probiotic therapeutics appropriate for clinical practice. In addition, sensitivity or intrinsic resistance of the majority of the strains to a recommended set of antibiotics make them safe for use in different products for human or animal consumption.
